# Ineffability: Reply to Professors Metz and Cooper

**DOI:** 10.1007/s11406-016-9772-1

**Published:** 2016-12-17

**Authors:** Guy Bennett-Hunter

**Affiliations:** University of Edinburgh, Edinburgh, UK

**Keywords:** David E. Cooper, Ineffability, Meaning, Meaning of life, Mystery, Philosophy of religion, Thaddeus Metz

## Abstract

In the first two sections of this reply article, I provide a brief introduction to the topic of ineffability and a summary of *Ineffability and Religious Experience*. This is followed, in section 3, by some reflections in reply to the response articles by Professors Metz and Cooper. Section 4 presents some concluding remarks on the future of philosophy of religion in the light of the most recent philosophical work on ineffability.

## Introduction

The concept of ineffability, which I define in terms of what is *in principle* resistant to conceptual grasp and literal linguistic articulation, has been an abiding presence in human thought for millennia. Yet this evanescent topic has been badly neglected by contemporary philosophy, whose focus has, for far too long, been on linguistic forms in isolation from the rest of life. The twentieth century saw the publication of a few important papers on ineffability in philosophy of religion (Alston 1956; Hick 2000). But it is only recently that renewed, sustained philosophical attention has been turned to ineffability per se (e.g., Cooper 2002; Bennett-Hunter 2014; Jonas 2016). This recent revival of interest is salutary, especially given the long history of this difficult topic. But some brief remarks on that history, whose context is chiefly religious thought, will make clear the profoundly ambiguous position that the concept of ineffability has continued to occupy.

The idea of ineffability has been at work in religious thought for many centuries, including the Christian tradition, with which I am most familiar—and by which my thoughts are mostly (but not exclusively) inspired. There have been many theological writers who, as Anthony Kenny points out, predicated their approach to theology on the concept of ineffability. Kenny (2006, 443) writes:

In centuries past theologians of unquestioned devoutness have maintained that God was ineffable, and indeed inconceivable. We humans, they maintained, cannot speak appropriately about God, and we cannot even think coherently about him. In a quite strict sense, it is impossible to use words about God. God is not something to be captured by human language.

However, writers in this vein, referred to as ‘apophatic’ or ‘negative’ theologians, have not always been of unquestioned devoutness. Indeed, two of the most influential examples, Pseudo-Dionysius the Areopagite (fl. c. 650–c. 725 ce) and Meister Eckhart (1260–1328), took up their positions at the fringes of Christian orthodoxy. Although they lived in very different historical periods (one in the classical world, the other in the medieval) both were influenced by neo-Platonism and both were regarded as heretical at some point in Christian history: Eckhart was condemned during his lifetime by the Roman Catholic Church, and Pseudo-Dionysius’s writings were regarded as orthodox until the fifteenth or sixteenth century. As Leszek Kołakowski (1988, 49) explains, regarding pseudo-Dionysius:

if the author of *On Divine Names* had not been mistaken for centuries for whom be pretended to be—the first Bishop of Athens converted by St Paul (Acts, 17.54)—he most probably would not have got away with his brazen neo-Platonism and his work would have remained in the annals of Christian though[t] as a heretical freak.

So despite its persistent centrality to religious thought, the concept of ineffability has always struggled to gain mainstream acceptance—a situation which persists in contemporary theology and philosophy of religion.

In an important article, which provides the first published description of the archived papers of the famous British philosopher of religion, John Hick, Thomas William Ruston (2016) provides evidence that, at least since 1940, Hick had been consistently interested, throughout his philosophical career, in the idea of an ineffable ultimate reality transcending all human categories, which he called ‘the Real’ or the ‘Transcategorial’. Interestingly, Ruston traces Hick’s source for the idea back to Spinoza, who was not exactly *persona grata* in theological circles. Ruston quotes a letter from the archives written by Hick’s friend and fellow philosopher, Peter Heath, who feared that, although Hick’s concept of ineffability appeared to open up a philosophical route to religious pluralism, his view would never gain widespread theological acceptance, and would even cause an ‘outcry’ against him.

There was certainly some precedent for such a fear, as Ruston (2016, 12, 13, 14–18) illustrates by describing two biographical episodes. In 1962, while at Princeton, Hick was put on ‘trial for heresy’ by the Presbyterian Church in the USA (of which he was also an ordained minister) and, in 1996, was criticized in a public address by Pope Emeritus Benedict XVI—then Cardinal Ratzinger. The first critique was widely known about and was even reported in the *New York Times*. And Pope Benedict’s critique determined the official position of the Roman Catholic Church on religious pluralism in general, even though, as Ruston’s article shows, his exposition of Hick was based on inaccurate scholarship. The substance of Pope Benedict’s complaint was that Hick’s concept of ineffability ruled out the exclusive truth of any human representation of the divine. But, as Ruston demonstrates, Hick’s view was that this simply follows from the view of divine ineffability that had been affirmed by mainstream, ‘orthodox’ theologians, including Gregory of Nyssa, Augustine, and Aquinas, as well as Pseudo-Dionysius, whose position, as we have seen, was less straightforward. But, despite the centrality of ineffability to the Christian tradition (as well as other religious traditions), the concept’s theological implications are still regarded as deeply ambiguous (Bennett-Hunter 2016b).

While some contemporary theologians and philosophers of religion pay lip service to ineffability (perhaps on account of its centrality to the work of the unquestionedly devout), most of them (unlike Hick) have been reluctant to follow through its logical implications for religion and theology. While they are happy to give formal assent to the idea of divine ineffability, such religious thinkers also invariably insist, for example, that ‘there are also reasons to think that the divine, the inner nature of which is linguistically inexpressible and incommunicable, makes itself known, however analogically, in ways that humans can partly comprehend’ (Ward 2016). However, when they are pressed on just what these reasons are, they tend, at best, to fall back on appeals to divine revelation, which, as Freud (2004, 31–2) pointed out, rely on circular reasoning. As I argue in my book (Bennett-Hunter 2014, 10–12 cf. Boyer 2007), even the most valiant philosophical attempts to analyse and defend the logic of this position are fundamentally confused. The main problem with such attempts, as I say, is that they fail to observe the crucial distinction between what is ineffable in principle (and of philosophical interest) and that which is so merely in practice, in a purely trivial sense.

It is against this turbulent background that *Ineffability and Religious Experience* endorses and develops David E. Cooper’s recent philosophical defence of the concept of ineffability per se and applies it systematically in philosophy of religion.

## *Précis* of Ineffability and Religious Experience

As Professor Cooper (2016, 000) points out in his article, there is a philosophical problem of ineffability that the book addresses: the tension between ineffability and answerability (Bennett-Hunter 2014, 14). This is the tension between the work that the concept of ineffability is required to do in order to be worth invoking and the concept itself. It is problematic to affirm a strong concept of the ineffable in principle and also to insist that there are ways in which human beings are answerable to the ineffable. While this tension exists in any appeal to the concept of ineffability, it is felt particularly keenly in religious contexts. As the theologian Brown (2008, 22) puts it, it is a tension that ‘exists in almost all forms of religion … that between explanation and mystery, between the conviction that something has been communicated by the divine (revelation) and the feeling that none the less God is infinitely beyond all our imaginings’. Indeed, this tension can be seen to be at work in the ongoing theological reluctance, just observed, to follow through ineffability’s logical implications.

To resolve this tension, I endorse Cooper’s recent argument for ineffability. The phenomenological background to his argument owes to ‘the thesis of the human world’: the humanistic view that any conceptualizable or articulable (i.e., ‘discursable’) world is a human world. He writes:

any world—physical and/or divine—that we could articulate and conceptualize is a ‘human world’: one that is the way it is only in relation to human perspective, purpose and preference, and not one, therefore, which is that way ‘in itself’ or ‘anyway’ independently of such human factors (Cooper 2005, 132).

But he views such humanism as impossibly raw and hubristic since it erroneously attributes to human beings the capacity to live with the sense that none of our concepts, values, beliefs, and commitments are answerable to anything beyond themselves, since there is nothing ‘beyond the human’. If we really believed this, Cooper argues, it could not have mattered to us if the concepts and meanings with which we invest the world, and the commitments and decisions that we happen to have made, had been different. This amounts to the belief that nothing is more or less worth believing or doing than anything else, a nihilistic belief with which Cooper does not think it genuinely possible to live. The fact that most of us do carry on with our lives as normal suggests that experience is not usually structured in this way. An apparent impasse results from the rejection of both ‘absolutism’ (the view that there is a discursable way the world is independently of the human contribution) and ‘uncompensated humanism’ (the view that there is nothing beyond the human which could provide measure for our lives). Cooper finds the resolution in an appeal to ineffability: the thought that ‘there is a way the world anyway and independently is, but that this way is not discursable’, in my terms, that ultimate reality is ineffable. According to this thought, Cooper (2009, 54) writes:

Absolutists … are right to insist that reality is independent of the human contribution, but wrong to suppose that this reality can be articulated. Humanists, correspondingly, are right to maintain that any discursable world is a human one, but wrong to equate reality with this world.

So the concept of ineffability involved is of a reality beyond the human that is, for that reason and in principle, ‘undiscursable’: unconceptualizable and literally inarticulable. This concept plays the strategic role of compensating for the humanist thesis without abandoning it, of providing measure for human Life:^1^ something beyond itself to which that Life can answer.

The argument thus far can be summarized in terms of the answer to a question regarding the meaning of Life. If the meanings of things, the concepts and values with which we invest them, must be explained in terms of their contribution to human concerns, practices, and projects—and therefore ultimately in terms of their relation of appropriateness to the human perspective, the world of human Life to which those practices and concerns themselves contribute—how can Life itself and as a whole be said to have meaning? The answer is: only by placing it in a relation of appropriateness to what is beyond itself, independent of the human contribution and therefore ultimately real. This ‘beyond’ cannot, without circularity, be invested with the concepts and meanings that constitute Life—which it is invoked to explain—therefore it must be ineffable.

But Cooper points out that, although it would abrogate the ineffability of ultimate reality to try to speak about it in literal terms, some kind of intimation of or attunement to it is required, to underwrite the view that certain ways of living are answerable to the way of things. He thinks that this requirement can be fulfilled by non-literal forms of language, ‘rhetorics’ and ‘poetries’ of ineffability found in the texts of various philosophical and religious traditions, which gesture towards what he calls ‘a sense or vision of the mysterious’ (Cooper 2009, 55). However, a very important point is that the requisite language does not encourage a disjunctive or dualistic vision of the relation of ineffable ultimate reality to the human world. Although the former must be thought of as ineffable and independent of the human perspective in order adequately to provide measure for it, it would be wrong to visualize it as disjoined from that world. In that case, we would be too prone to envisage the ineffable in absolutist terms, for example, as a transcendent Kantian realm or a thing, like a cosmos or (for Cooper) a god: no less discursable than the human world itself because invested with (at least some of) the very concepts and meanings for which it was supposed to provide measure—precisely by not being so invested.

John Hick’s (2000, 40) neo-Kantian vision of the ineffable (which he sometimes refers to in religious terms) as a ‘joint product of [the divine] presence and our own conceptual systems and their associated spiritual practices’ falls into this error and, from Cooper’s viewpoint, is incoherent. For, as Cooper points out, it allows a very major exception to the general claim that the world depends for what it is on human beings (for example, on the a priori structures of the mind), and that is human existence itself. The question is then raised, what place we ourselves could have in such a world, so much of our own making. This view requires us, impossibly, to be, ‘so to speak, … already there, up and running, … responsible for the world taking on the contours it does’ (Cooper 2005, 134).

So Cooper’s solution is a non-literal vocabulary that encourages a vision of intimacy between the human world and its ineffable measure or ‘source’: a transformed attitude of attunement towards the human world itself. Following the vocabulary of the later Heidegger, he suggests that we should think of the human world as the ‘epiphanizing’ or ‘presencing’ of ultimate reality—as a mysterious ‘gift’. ‘This world,’ as Cooper (2009, 58) writes, ‘is not simply a human world unthinkable in isolation from us, but at the same time a realization of, a coming forth of, something to which we can strive to answer and measure up.’ His argument for the ineffability of ultimate reality, then, is intended to provide a kind of answer to the question of the meaning of Life. Its existential phenomenological approach, which differently construes the nature of the relationship between human beings and the world (and between the human world and its ineffable ‘measure’), allows it to answer this question in a way that resolves the tension between ineffability and answerability—which, my view, also provides a way of surmounting the main obstacle to making sense of ineffability in philosophy of religion: the problem of the possibility of experiencing an ineffable God.

In response to the question how it is possible to secure reference to what is in principle ineffable, I respond that the distinction is observed between language that talks about things and that which talks about language. I suggest that the word ‘God’ is understood in parallel to other philosophical terms like ‘ultimate reality’, ‘the absolute’, ‘Being’, and ‘Transcendence’ to refer to the concept of ineffability and thereby to evoke (but obviously not refer to, describe, or express) the ineffable. While Cooper is reluctant to use theistic terminology when invoking his notion of ineffability, the language of divinity and its associated ritual practices may turn out to be particularly effective means of evoking the transparency that Cooper mentions of the human world to its ineffable measure. The word ‘God’ and its cognates are already used by many religious people to evoke what is ultimately real, is appealed to in religious explanations of the meaning of human life, and which religious practices properly intend. Indeed, Martin Buber recounts a conversation in which a friend suggested to him that it seems almost blasphemous to use the word ‘God’ for the highest ultimate reality, because that reality is thereby lowered to human conceptualization. Buber responded that the word can be so evocative precisely because it has become so ‘soiled’ and ‘mutilated’. He writes, ‘Just for this reason I may not abandon it. Generations of [women and] men have laid the burden of their anxious lives upon this word and weighed it to the ground; it lies in the dust and bears their whole burden. … Where might I find a word like it to describe the highest!’ (Buber 1953, 16–17). Admittedly, many religious uses of ‘God’ carry what John Cottingham (2006) calls ‘doxastic freight’: literally understood, their content looks incompatible with the concept of ineffability. But apophatic theologians have, for centuries, been treating that freight as just so much ballast to be jettisoned. Language is dynamic and continuously subject to interpretation and reinterpretation. It is therefore surely mistaken to assume that all uses of the language of divinity—and still less all religious practices—necessarily imply concepts and attitudes that are incompatible with the concept of ineffability, as they often do on a purely literal interpretation.

On the basis of considerations such as these, the last three chapters of my book apply Cooper’s philosophical approach to ineffability in the context of philosophy of religion, developing a new philosophical account of divine ineffability, which also draws on the unjustly neglected philosophy of Karl Jaspers. While it will be impossible to describe this account fully in the space available, in what follows, I draw attention to the theological implications of the concept of ineffability that are of most relevance to the response articles by Metz and Cooper.

In order to avoid the familiar self-reference antinomy apparently involved in ineffability claims (see, e.g., Kołakowski 1988, 44), I suggest that the word ‘God’ be understood as a religious reference (alongside parallel philosophical terms), not to the ineffable but to the concept of ineffability. These terms thereby evoke, but obviously do not describe or express, the ineffable. An important implication, as just intimated, is that ontotheology (the idea that the word ‘God’ refers to something that exists, in the same way that objects or we ourselves exist) should be rejected. ‘God’ can be understood to refer to the concept of what explains the meanings and concepts of the human world, and therefore the meaning of human Life, only by not being invested with those concepts and meanings. And, from the perspectives of phenomenology and contemporary pragmatism by which I am influenced here, the concept of existence must be included among these. From the phenomenological perspective, Kołakowski (1975, 65) reads Husserl as follows:

“Existence” itself is a certain “sense” of an object. Consequently it would be absurd … to say that an object “exists” independently of the meaning of the word “to exist”—independently of the act of constitution performed by the consciousness.

As the Finnish pragmatist philosopher, Sami Pihlström (1996, 123) puts it, ‘Words like “object” and “exists” are used in different ways in different contexts (or language-games). No usage is “forced” by the world.’ And William James (1922, 242) makes the point in broader terms: ‘to an unascertainable extent,’ he writes, ‘our truths are man-made products’. For all three thinkers, the human world is shaped and determined by our human interests, perspectives, and practices—and, certainly for the first two, this necessarily includes our most basic ontological categories.

If Cooper is correct, as I think he is, then the concept of ineffability is the *only* concept that one can appeal to in order to evoke the ultimately real and therefore explain the meaning of life without circularity. And the denial that any concepts apply to the ineffable, must extend to the concept of existence. In theological terms, if the word ‘God’ is taken to refer to the concept of what accounts for the meaning of everything that exists, it cannot also be thought of as referring to one of the things that exist. Some twentieth-century and contemporary theologians who have intuited this point have revisited the mystical tradition,^2^ carrying out thoroughgoing critiques of ontotheology, in the light of which we can understand, for example, Meister Eckhart’s refusal to mention ‘God’ and ‘existence’ in the same theological breath: Eckhart controversially oscillated between the denial that God exists and the opposite claim that *only* God exists. Such theologians have intuited an important theological implication of the concept of ineffability: that the word ‘God’ can be understood as a reference to the concept of ineffable ultimate reality only if we give up the reassuring thought that, in any familiar sense, ‘God exists’. We must either radically revise the concept of existence (which seems otiose and otherwise unnecessary) or give up the idea that, properly understood, the word ‘God’ refers to an entity. This theological line of thought finds philosophical support in the phenomenological approach that has remained relatively undeveloped in philosophy of religion in general.

A second implication is that, in order to contemplate the possibility of religious experience after the rejection of ontotheology, we will have to be very clear just what is meant by ‘experience’. I agree with Cooper (1985) that, at least in this demesne, it is unreasonable to demand that ‘experience’ has to involve the application of concepts, which would be incompatible with ineffability; I agree that the distinction between subjective and objective dimensions to the notion of experience may be ‘badly drawn’; and I also agree with phenomenological and pragmatist critiques—notably, John Dewey’s (1949, 187)—of the dualistic construal of the subject-object distinction as a dichotomy, which renders the potential relationship between the distinguished items unintelligible. With regard to the concept of experience, such a dualistic understanding entails the vagueness and ambiguity in the phrase ‘what I experienced’, which could refer equally to the object of an experience as to the experience itself. So, in order to understand the nature of religious experience in the light of divine ineffability, we have to consider a very different understanding of experience: one that does not require the application of concepts nor a dualistic distinction between its subjective and objective dimensions.

While some theologians who are sympathetic to divine ineffability have tried to account for religious experience (and expression) in symbolic terms, I argue in my book that this concept of symbol ends up reinforcing an unhelpful dichotomy between subject and object (Bennett-Hunter 2014, 67–75). I find an alternative, and more congenial, concept to be Karl Jaspers’s ‘ciphers [*Chiffren*]’. Whereas symbols are typically understood as objective realities that intend other realities (whether or not they exist outside the symbol) and objectify them in the symbolic representation, ciphers, for Jaspers, are irreducible to either pole of the distinction. They are understood as subjective and objective at once and hence like the ‘language’ or ‘physiognomy’ of the ineffable reality that can be experienced only in and through the cipher. Therefore, while a symbol can only symbolize something *within* the subject–object distinction, a cipher can embody what Jaspers calls ‘Transcendence or God’, by which he means the ineffable ultimate reality that is unconditioned by that distinction—and, from my anti-ontotheological perspective, is certainly irreducible to its objective pole. Although I take issue with some of the detail of Jaspers’s account of ciphers (especially in the context of his complex and monumental philosophical system as a whole), I argue in my book that, on this basis, religious experience should be interpreted as the experience of religious ciphers: parts of the (natural or cultural) human world that are experienced as transparent to, or intimate with, the ineffable, divine source of that intractably meaningful world. And although I cannot go into this in any detail here, I also think that religious *expression* should be interpreted, in the light of divine ineffability, as a set of linguistic and non-linguistic practices that both reflect and bring about this kind of experiential attunement of oneself and others to the ineffable. I argue for four parallels between religious and aesthetic expression, drawing on the phenomenology of art and the later Wittgenstein, to make the case that, after the rejection of ontotheology, religious language and ritual practices are not best understood as obviously bad attempts to describe the ‘religious object’ experienced (a notion which begins to look incoherent from my anti-ontotheological point of view) but rather as evocations of what cannot be described: through the *way* in which a story is told and the *manner* in which a ritual is performed, rather than primarily through their cognitive content (Bennett-Hunter 2014, 133–148).

Here, I expand briefly on my account of religious ciphers by contrasting it with the religious pluralism for which John Hick became notorious. For Hick, religious pluralism follows from divine ineffability because of the Kantian distinction that (as we saw earlier) he applies in the religious demesne between the ineffable, noumenal Real *an sich* and its phenomenal appearances to human perceivers. Since the latter vary from religion to religion, Hick concludes a principle of equal validity between the world’s religions: the idea, as he puts it, ‘that human religious experience is a range of responses to a transcendent reality, taken together with the observation that the moral and spiritual fruits of the different world faiths are, so far as we can tell, equally valuable’ (Hick 2000, 41). I have already explained my reasons for finding Hick’s neo-Kantian perspective on ineffability questionable. But, in a religious context, the necessary rejection of ontotheology further problematizes Hick’s sharp distinction between the noumenal, ineffable divine reality and its phenomenal appearances as well as his ultimately disjunctive vision of their relationship. Hick’s pluralism acknowledges the formal differences between religions, which are explained in terms of differences in the Real’s phenomenal appearance in different cultures and religions. But what makes religions equally valuable, in Hick’s view, is their status as different responses to the *same* divine reality, experienced in correspondingly different ways. For Hick, all religions are equally true, insofar as they evoke, or respond to, the same ineffable, divine object. In my view, it is clear that Hick has not followed the idea of divine ineffability to its logical conclusion: the rejection of ontotheology. Hick’s God is still an object, invested with the concept of existence, and therefore at least minimally conceptualizable. So I question whether Hick is *really* committed to divine ineffability, in my sense.

My own position, by contrast, is based on a neo-Jaspersian view, according to which religious experience is construed as a process of cipher-reading. Also influenced by Kant, Karl Jaspers speaks of the ‘phenomenality’ of the human world but denies that there is anything noumenal with which it might be contrasted. Since, *qua* ineffable, ‘Transcendence or God’, is not an object, it is discernible for us at all only in and through our reading of the cipher-script of that phenomenal world itself. Just as a person’s being is perceptible in their ‘physiognomy’ and ‘involuntary gestures’, Jaspers (1969a, vol. 3, 124–125) says, so we experience the physiognomy, as it were, of all existence. Whereas human physiognomy concerns the expression of something accessible in other ways (by empirical psychology, for example), Transcendence is accessible only in and through that ‘physiognomy’. Jaspers (1969a, vol. 3, 134) elaborates this transparent view of the phenomenal world:

This transparent view of existence is like a physiognomic viewing—but not like the bad physiognomy aimed a form of knowledge, with inferences drawn, from signs, on something underneath; it is like the true physiognomy whose ‘knowledge’ is all in the viewing.

Rejecting the idea of any determinate reality *to* which the world becomes transparent, Jaspers (1967, 7) is clear that Transcendence or God is a boundary concept, rather than an object, and that to our cognition, it indicates nothing beyond our imprisonment in phenomenal appearance. But when we read the phenomenal world as a cipher-script of Transcendence, he writes:

The phenomena in the dichotomy [*der Spaltung*] grow brighter. We sense the encompassing [i.e., reality including its transcendent mode] in them. The prison walls are not toppled … but if we know the prison, if we see it from the outside as well, so to speak, the walls become transparent[, which …] makes the jail less and less of a jail (Jaspers 1967, 79).

In common with Hick, this view of our being imprisoned in appearance implies that no human perspective on reality (including religious ones) could ever be completely and exclusively true. But it does not amount to Hick’s bolder, pluralistic claim that all religions are *equally* true because they are apprehensions of the same noumenal object. Considering the relationship between ciphers and religious traditions, Jaspers consistently denies that there can be a definitive system of ciphers, although he allows that a system can itself be a cipher among others. He says:

*From where I stand*, the cipher remains permanently ambiguous—which means, speaking from the standpoint of transcendence, that transcendence *has other ways yet to convey itself* (Jaspers 1969a, vol. 3, 131).

So if we think, in Jaspersian terms, of religious systems as ciphers of Transcendence or God, what matters is not the tradition’s concrete forms but the way in which these are understood (or ‘read’) philosophically, which is why philosophy becomes an essential companion, even prerequisite, to religion and theology from Jaspers’s point of view. For example, if the religious system of Christianity were read as a cipher, its

Dogmas, sacraments, rituals would be melted down, so to speak—not destroyed, but given other forms of conscious realization.… Not the substance, but the appearance in consciousness would change. Philosophy and theology would be on the road to reunification (Jaspers 1967, 340).

If Jaspers is right that any part of the world, or even the world as a whole, can become transparent to the ineffable, a cipher of Transcendence, then no determinate meaning, religious or non-religious, should in principle be debarred as a valid imagining or evocation of the ineffable. In his view, what he calls the ‘poison’ of exclusive claims has to be removed in order for religious experience to be ‘melted down’ into reading of a cipher:

Removing the poison consciously takes a simple and momentous insight: that exact, generally valid truth is relative, dependent on premises and methods of cognition but compelling for every intellect, while existential truth is historic, absolute in each [person’s] life but not to be stated as valid for … all others (Jaspers 1967, 340).

Rather than deserving the heated controversy that Hick faced, it should be recognized that such an inclusive perspective (though far from Hick’s ‘pluralism’) follows from the notion of divine ineffability, in the sense that this has been operative, in the Christian tradition, since at least the sixth or seventh century ce. What will be most important for the future of philosophy of religion is the kind of continuing conversation that ineffability could make possible between members of different religious traditions, both theistic and non-theistic — and, by extension, between religious people and secular atheists. I return to the implications of this important point for further research in the discipline in the final section of this article.

In summary, then, the main aim of *Ineffability and Religious Experience* is to situate the phenomenology of religious phenomena, most importantly the various linguistic and non-linguistic forms of religious praxis, within a philosophical framework to which the concept of ineffability is central. This new philosophical theory of divine ineffability draws on Karl Jaspers’s philosophy to construe the experience of such religious phenomena as a process of cipher-reading: a transformed attitude of attunement to parts of the (natural or cultural) human world itself, or to that world as a whole. Such a reading reveals the world’s total intimacy with its ‘ground’, ‘measure’, or ‘source’—one appropriate name for which is ‘God’. This theory implies the rejection of two thoughts (1) the ontotheological idea that ‘God exists’ and (2) that any human perspective on, or representation of, (divine) reality could be exhaustively or exclusively true. Given the theological popularity of these two thoughts, the implications of such a theory may be difficult for some theologians to accept, but no coherent way of thinking about ineffability in a religious context is able to avoid these implications. Moreover, as I started out by noting, despite its ubiquity, the concept of ineffability has always struggled to gain mainstream theological acceptance.

## Reply to Professors Metz and Cooper

### Reply to Professor Metz

In his perceptive response article, Professor Metz correctly locates the foundations of my project in a search for ultimate meaning—meaning (to repeat Nozick’s useful phrase) ‘all the way down’. Metz rightly observes that I understand meaning in relational terms. Following Cooper (2003, 2005), my view is that to explain the meaning of something is to specify its relation of appropriateness to a context broader than itself, ultimately to the context of human Life.^3^ I agree with Cooper’s (2005, 126) initial definition of meaning as ‘appropriateness to Life’, and his appeal to ineffability as enabling the only convincing answer to the question of the meaning of Life itself, construed as the terminus of explanations of meaning or, to put it another way, the source of ultimate meaning. But, in this connection, the most relevant implication of the concept of ineffability is the fact that it shipwrecks the subject–object distinction. It evokes a reality that is not an object that could possibly be comprehended by a subject (cf. Heidegger 1962, 89), thereby demonstrating the limits of the subject–object distinction and falsifying dualistic construals of that distinction as a dichotomy. A full reply to Metz’s worries about my appeal to ineffability as the source of ultimate meaning will require some further elaboration of this thought.

In my book (Bennett-Hunter 2014, ch. 5), I provide a supplementary reading of the rational status of Cooper’s argument for ineffability, guided by the recent work of Iain McGilchrist (2009, ch. 4). In order to be rational, an argument has to presuppose the validity of the subject–object distinction. But what is the rational status of an argument which concludes to a concept that shipwrecks that distinction? Briefly, my answer is that the argument makes initial use of reason in order rationally to demonstrate both reason’s limitations and the necessity of a form of discourse that is not wholly rational (like Cooper’s ‘rhetorics’ and ‘poetries’), which is unconditioned by the subject–object distinction and whereby the ineffable may therefore be evoked. This argument presents us with a picture of reason as being like a ladder, which, once climbed (and *only* then), must be kicked away (cf. Wittgenstein 1988, §6.54). It is a rational argument that defends a phenomenological perspective, from which the world ‘reason’ no longer signifies anything specific that is wholly distinct from what is signified by the word ‘passion’, for example. From this phenomenological perspective there seems little point in speaking of a ‘purely rational’ dimension to human existence. From this perspective, there is a palpable demand for a kind of philosophical discourse which, in line with the phenomena in which experienced reality consists, inextricably includes what are often abstractly separated as ‘rational’, ‘evaluative’, and ‘affective’ dimensions.

In my view, reflection on the rational status of Cooper’s argument for ineffability reveals something important about the nature of existential phenomenology: that, as a philosophical method with a tendency to dissolve dualisms (Cooper 1999, ch. 5), it incorporates both a rational and a descriptive/poetic pole, effecting a dialectic between them. (I call this the ‘phenomenological dialectic’ (Bennett-Hunter 2014, 123–131)). Cooper’s argument begins with a rational approach, which assumes the validity of the subject–object distinction, but that argument concludes to a concept (that of ineffability) that eludes further meaningful articulation in the rational terms of that distinction. Cooper (2005, 137) is clear that, to avoid an incoherent neo-Kantian perspective, the appeal to ineffability involves a feat of ‘double exposure’, which simultaneously reveals human Life as both that to which meanings ultimately answer and, if it is itself to be experienced as meaningful (answerable or measurable), as the coming to presence of the ineffable beyond the human. This human world is revealed by such a double exposure as ‘not simply a human world unthinkable in isolation from us, but at the same time a realization of, a coming forth of, something to which we can strive to answer and measure up’ (Cooper 2009, 58). It is here that the rational terms in which Cooper’s argument is initially presented begin to give way. He ends his 2005 article on ‘Life and Meaning’ with the following words, which perhaps read like an understatement:

The delicacy of performing this feat of ‘double exposure’ should not be in question. So it is not very surprising, perhaps, that I find myself drawn, in my two claims, to speak in ways that are difficult simultaneously to combine. (Cooper 2005, 137)

*Pace* Sartre, it would be philosophically irresponsible to indulge in the descriptive poeticising for which this argument calls *before* reason’s limitations have been established *on rational grounds* (Bennett-Hunter 2014, 107–11). The ladder must be climbed before it can be kicked away. At its best, as McGilchrist observes, existential phenomenology does not consist in an arbitrary preference for, and turn towards, experience over logic. Phenomenology begins with the conventional philosophical tools: clarity of thought and expression, a striving towards precision, consistency, and cogency of argument. At its best, it represents the distinctive philosophical impulse to transcend the usual, purely rational scope of philosophical inquiry. Its rational pole (literalistic, explicit, and analytical) is complemented by its descriptive and poetic pole, which pays attention to reality precisely as it is experienced. As Jaspers (1956, 113–7) and McGilchrist (2009, 140) point out in their lucid readings of some of the famous pre-Socratic paradoxes, such description could not be carried out in purely rational terms without contradiction. Perhaps appropriately, McGilchrist (2009, 135) describes the distinctive philosophical impulse, found in phenomenology and pragmatism, to transcend the demesne of the purely rational with the aid of a bizarre simile:

Admittedly, trying to achieve it at all using the conventional tools of philosophy would be a bit like trying to fly using a submarine, all the while making ingenious adaptations to the design to enable one to get a foot or two above the water. The odds against success would be huge, but the attempt alone would be indicative that there was something compelling beyond the normal terms of reference, that forced one to make the attempt.

It is in these terms that I explain the tendency of some existential phenomenologists to speak of an ultimate ‘source’ of the human world, or a ‘mystery’ to which it answers (see, e.g., Marcel 1948). These philosophers are striving towards a source of ultimate meaning for the human world, a terminus of explanations of the meaning of Life, which, for that very reason, cannot be exhaustively captured in the rational terms in which all philosophy quite properly begins. And it will be clear why I think it unsurprising that some of these writers find religious or quasi-religious significance in this idea. Philosophers impossibly committed to rationality (by which I mean a particular understanding of reason that presupposes a dualistic distinction between subject and object and which thereby leaves open the possibility of reason’s in principle *unlimited* operation in isolation from other forms of human engagement with the world (Bennett-Hunter 2014, 112)) will complain that the descriptive and poetic discourse in which phenomenology often results is simply not philosophy. But, if my reading of the rational status of Cooper’s argument is well taken, the phenomenologist must reply that it is what philosophy, even as these philosophical opponents understand it, necessarily becomes. It is the consummation of philosophy, when philosophy takes reason as its guiding light in the absence of any other, when it follows through the implications of its own logical operations. Itself still philosophy, it is no longer mere rationality but has become the consummation of reason. On the other hand, it would be easy to draw the mistaken conclusion that because existential phenomenology is often poetic and challenges the idea that reason has an exhaustive scope, it does not itself involve reason. The phenomenologist must answer that her method does involve reason, but only to the limited extent that reason itself permits. It does so to the extent, firstly, that within the terms of the subject–object *distinction*, it may defend its own methodology in opposition to rationality’s *dichotomy* between subject and object and, secondly, that it might show that its own consummation in a poetry of attunement to the ineffable takes place, not as an arbitrary substitute to reason but only with reason’s shipwreck. As a more or less explicit intuition, this central thought is present as an abiding theme in Karl Jaspers’s (1956, 119) philosophy:^4^

That the whole of my rationality rests upon the basis of non-reason—such a phrase does not assert that reason can be denied out of some general right drawn from existential philosophy. Nothing which lacks reason or which is contrary to reason can raise up argumentative claims out of itself, for precisely this process enters into the medium of rationality. … Every premise of justification enters into the medium of the rational. The truth of the non-rational is impossible unless reason is pushed to its limit.

One of Metz’s concerns is that the concept of ineffability cannot terminate the chain of questioning regarding meaning and that, even if we assume that it can, we could never know whether our lives were answerable to the ineffable or not. His concern is that ‘my inability to refer to (or know) the relevant object’ would prevent me from explaining the meaning of Life in terms of it, describing my view of the human condition as ‘like being in an unavoidably dark room and told to attune myself to the work of art on the wall’ (Metz 2016, 000). In Kantian terms, my suggestion looks to Metz like an instruction to comprehend an appearance by appealing to the thing-in-itself. My reply to the worry concerning reference is to repeat the point that, although one cannot refer to the ineffable, one can evoke the ineffable by referring to the concept of ineffability: for me, the ineffable is not an object at all and, as Metz observes, is a reality toward which we can *only* gesture. Therefore the language of ‘measure’, to which Metz (2016, 000) objects, must be understood in non-literal terms. Unlike some recent writers on ineffability (Jonas 2016, 121–127), my view is that there can be irreducibly metaphorical, or otherwise non-literal, forms of language. But these forms of language, which gather at the descriptive, poetic pole of phenomenology are only licensed once the literal, rational forms have been exhausted by being pushed to their limits. So, as I have been suggesting, although the validity of the concept of ineffability can be rationally demonstrated, its content (or rather its contentlessness) cannot be further articulated in purely cognitive, rational terms. It is the shipwreck of the subject–object distinction that licenses the construal of attunement to the ineffable as a process of cipher-reading, a transformed attitude of attunement towards our human world itself that can be both evoked and cultivated in arational, poetic terms. With Jaspers, who writes of our ‘imprisonment’ in appearance, I, too, write about the ‘phenomenality’ of the human world, but deny that there is any determinate noumenal ‘object’ with which the phenomenal might be contrasted. Jaspers (1969a, vol. 1, 82) describes the intellectual movement of transcending as one that ‘leaves the world but does not lead out of the world to something else’; its meaning is derived from the boundary concept, not from an object (to which it would be possible to secure reference), nor from an alternative world of objects. Therefore, in my view, the ineffable is not best evoked as being like a work of art on the wall of an unavoidably dark room but rather as being like rays of light which potentially illuminate all phenomena and allow them to be experienced in new ways, but those rays of light have no objective, (‘noumenal’) source beyond that human world itself. For this reason, I am sympathetic to Cooper’s (2002, 327 cf. 2006, ch. 7) language of ‘epiphanies’ of the ineffable; he points out that the phrase ‘epiphany of…’ is more akin to ‘flash of lighting’ than ‘flash of a knife’, i.e., an expression ‘where it would obviously be mistaken to imagine a divide between what shows and its showing’. When attuned to the ineffable measure of the human world, when answerable to the ineffable, my experience is of what Jaspers calls the ‘physiognomy’ of existence. So in answer to Metz’s question how I could ever know that my Life was answerable to the ineffable, I give Jaspers’s (1969a, vol. 3, 134) reply: that this is ‘not like the bad physiognomy aimed a form of knowledge, with inferences drawn, from signs, on something underneath; it is like the true physiognomy whose “knowledge” is all in the viewing’. In other words, the question is rendered illegitimate by the shipwreck of the subject–object distinction, after which narrow, cognitive interpretations of the nature and significance of the experience are manifestly inappropriate.

Another concern that Metz raises is with my view that meaning is exclusively relational. Could we not rather say that ‘while *some* meaning can accrue in virtue of properties intrinsic to a person, *more* meaning would accrue insofar as those properties were to relate to broader contexts’ (Metz 2016, 000)? In Metz’s view, apparently, meaning is a ‘thick’ concept: when one explains something’s meaning, one will be able to appeal to objective properties of that thing in virtue of which it is to be judged meaningful. My problem with this owes to my existential phenomenological perspective, according to which meaning is not a thick concept but something more like the ‘thickness’ of concepts: the condition for the application of concepts and for the carving up of lived experience into subjects (which apply concepts) and objects (to which concepts are applied). To be sure, we can appeal to objective features of, say, flour (its propensity to rise when baked) in order to explain a meaning that it has in human Life (an essential ingredient of bread). But to an unascertainable extent those objective features owe to the subjective interests, perspectives, and practices that have led us human beings to carve the world of experience up in the way that we have in the first place (e.g., bread, its ingredients, and their respective roles in our lives). I agree with Putnam’s neo-pragmatist expression of this point: ‘A being with no values would have no facts either.’ (Putnam, cited in Pihlström 1996, 274).

I am even less sanguine about the feasibility of the conception of meaning as a thick concept in the context of explanations of the meaning of Life as a whole. For, in that case, pointing to certain objective features of Life itself could be sufficient for an explanation (a partial one, at least) of its meaning; in other words, Life would have at least *some* inherent meaning. In his recent book, James Tartaglia (2016, 43) compares life to a game of chess and the idea that life has meaning (construed as a relation to something beyond itself) to the possibility of achieving checkmate, which may motivate a person’s moves in the game. But Tartaglia believes that checkmate is an unavailable illusion: there is nothing beyond life in terms of which its meaning could be explained. Therefore, he counsels us to focus instead on the moves in the game themselves. Having discovered that these are, in fact, ‘the only real goals’, we must value them ‘for their own sake’. In other words, the only kind of meaning that we will be able to find for Life will be inherent in Life itself. In my view, however, the unavailability of checkmate would undermine the meaning of the moves in the game of chess, which have no ‘inherent meaning’ for which they might be valued ‘for their own sake’: their meaning just consists in their contribution to the possibility of checkmate. My view is supported by the fact that Tartaglia’s book is, in fact, an argument for nihilism: his main conclusion is not that Life is partially meaningful, but that, given the absence of a transcendent context of meaning, Life, together with the whole of reality, is wholly meaning*less*. If Tartaglia really believed that there were such a thing as ‘inherent meaning’ that it were possible to find within human Life, he would have reached a different conclusion (see Bennett-Hunter 2016a).

But while I cannot commit to the letter of Metz’s alternative proposal, I am sympathetic to the spirit: that in one, importantly qualified, sense, Life itself is what ultimately supplies meaning. Metz (2016, 000) concludes with the suggestion that ‘[s]eeking meaning “all the way down” could lead to a spade being turned upon the earthly, the comprehensible, the finite’. I have no quarrel with this conclusion but, in the light of Jaspers’s remarks on cipher-reading, I want to add that the quest for ultimate meaning requires an ability to experience the finite *in a new way*, with a transparency to the ineffable reality with which, in the appropriate mode of attunement, the finite world is experienced as being wholly intimate. However, it will be clear from my previous remarks that this concession can be made only after the subject–object distinction has been tested to its limits. Once again, the ladder has to be climbed before it can be kicked away.

### Reply to Professor Cooper

Professor Cooper’s response article sensitively reflects on music, nature, and their relationship to support my suggestion that art (and religious rituals) may cultivate a sense of ineffability, ‘body it forth’, and enable it to do the kind of work in human life that I have been describing. Both art and ritual have their strengths and weaknesses in carrying out this important role. Works of art often seem disconnected from the rest of human life, sealed off from its quotidian realities, bounded by frames, locked up and carefully guarded in museums and galleries. But they are less often subject to the same kind of dogmatism that is attendant upon many religious rituals. No doubt there are ‘dogmatic’ curators and art critics but thankfully such people do not have the kind of finely-grained control over the lives of ordinary people that is wielded by some religious leaders. Conversely, the dangerous potential for dogmatism notwithstanding, religious rituals are typically much more imbricated with the fabric of people’s lives than most works of art. As John Cottingham (2003, 98) points out, the repetitive, rhythmic pattern of daily ritual practices, like saying morning and evening prayer and grace before meals, allows the otherwise mundane rhythm of eating and sleeping to take on a religious significance. The shape of a life as a whole, too, may be religiously structured, in some traditions, by the ritual structure of the liturgical year and, perhaps universally, by the marking off of significant life events by rites of passage: birth by baptism, copulation by marriage, and death by cremation or burial. One of the advantages of Cooper’s stress, in his response article, on the ‘complete resistance’ to a separation between the natural and the cultural is that it allows for a more intimate connection between works of art and the rest of life. Cooper’s (2006) remarks on gardens (neither wholly ‘art’ nor wholly ‘nature’) as places where such opposites can be reconciled and ‘epiphanies’ of a life attuned to the ineffable can be experienced point in this direction. Also relevant is the ritual significance that some gardens, notably Zen gardens, have (Cooper 2006, 118). Similarly, his remarks on the creative, intimate, and mutually informing relationships between music like John Cage’s and the ‘ambient sounds’ of the rest of life, including the natural world, suggest the exciting possibility that at least some works of art invite the kind of experience that attends to a more intimate relationship with the natural environment and with the rest of life. The exciting possibility opened up by Cooper’s (2016, 000) remarks is for some aesthetic experiences to be atypically ‘skilful means’ of attunement to the ineffable.

One of Cooper’s (2016, 000) concerns is with my claim that works of art are ineffable because their meaning is inexhaustible; ‘it sounds strange’, he writes, ‘to regard this as a reason for calling the work “ineffable”. Usually, we think of something as ineffable because description of it can never begin, not because it can never end.’ My reply to this objection is that, in my view, works of art do not describe but rather evoke the ineffable. To return to phenomenology’s rational and poetic poles, while rational discussion of the ineffable can never start, its poetic evocation can never stop. This is because, as we heard Jaspers insisting, no human imagining of the ineffable could ever be exhaustive or definitive. However, I concede Cooper’s (2016, 000) point that it ‘cannot be for this reason, or in this sense, that art works, which are describable, are ineffable’. What is ineffable is the *meaning* of the work: the ‘real presences’ that, for Merleau-Ponty (1964, 162) and George Steiner, the artist embodies in her work, effecting ‘transbstantiations’ of the ineffable into pigment or some other artistic medium (Bennett-Hunter 2014, 147–8).^5^

In his response article, Cooper helpfully emphasizes the broad nature of my concept of experience. This, too, is attributable to the shipwreck of the subject–object distinction on the concept of ineffability. Drawing here on some of Cooper’s (1985) earlier work, as well as Jaspers’s account of ciphers, I find the distinction between experience’s subjective and objective dimensions to be signally unhelpful in this demesne. After the rejection of ontotheology, religious experiences of the ineffable are not of any ‘object’ at all, neither can they be understood in purely cognitive terms. My concept of experience is one that does not require the application of concepts (which would entail that the ineffable could not, by definition, be experienced) nor, as I explained earlier, a dualistic distinction between its subjective and objective dimensions. This is why I prefer Jaspers’s theory of ciphers to theories of religious symbols. Ciphers are irreducible to either pole of the subject–object distinction; their purpose is to enable us to transcend the distinction. Jaspers (1969b, 93–94) writes:

Ciphers are objective: in them something is heard that comes to meet man. Ciphers are subjective: man creates them by his way of apprehending, his way of thinking, his powers of conception. In the subject–object dichotomy [*Spaltung*] ciphers are subjective and objective at once.

Finally, in the limited space available, Cooper is reluctant to go into detail about ‘[w]hat, exactly, a life is like that is led in a manner answerable to a sense of mystery’ but the foregoing remarks suggest, in harmony with Metz’s view about what gives Life meaning, that it would, at the very least, involve renewed attention to the quotidian fabric of that Life itself. While no literary portrait could, by definition, be definitive, Hugo von Hofmannsthal’s (2005) ‘The Lord Chandos Letter’ may be a helpful place to start. As Cooper’s response article stresses, I have argued that art may be understood as a powerful means of cultivating such answerability and a rich source of ciphers—as may linguistic and non-linguistic religious practices, when the cipher is read in the responsible, philosophical way that Jaspers prescribed. Recall that, while Jaspers (1969a, vol. 3, 131) rejects the idea that any system could be a definitive system of ciphers, he allows that ‘a system can itself be a cipher’. In my view, religious systems should be viewed, like works of art, as ciphers of the ineffable.

Jaspers was hopeful that, if religious experience and expression were ‘melted down’ into a process of cipher-reading and the ‘poison’ of exclusive claims removed from religious discourse, philosophy and theology would be on the path to reunification. I share Karl Jaspers’s sense of hope and conclude this article with some suitably tentative remarks on the direction that future work towards such reunification might take.

## The Future of Ineffability: Remarks on Further Research in Philosophy of Religion

In this article, I have presented my reading of Cooper’s argument for ineffability as a rational argument that demonstrates reason’s limitations and reveals phenomenology as a philosophical method, with rational and poetic poles, which enables philosophy’s, usually purely rational, self-imposed limits to be transcended. I compared this argument with a ladder, which, once it has been ascended, must be kicked away. But while the reader might accept the idea that there may be a point at the top of the ladder, where, on pain of irrationality, reason must be abandoned, they may be wondering about the foundations of reason. How solid is the material into which the bottom of the ladder is sunk? Wittgenstein’s (1969) last work, *On Certainty* (which was never prepared or sanctioned for publication by Wittgenstein himself), sheds some light on this question. For Wittgenstein (as is well known), there are ‘hinges’ on which *any* epistemic evaluation must turn. If rational inquiry is to get off the ground at all, certain propositions have to be assumed and taken for granted. Following the non-epistemic reading offered by Duncan Pritchard (2012), these hinge commitments, which Wittgenstein describes as having the character of indubitable certainty, are essentially arational. Just as one cannot rationally doubt a hinge proposition, one cannot rationally believe it either (Pritchard 2012, 257). To have arational hinge commitments, for Wittgenstein, is just what it is to be rational. This Wittgensteinian picture of the structure of reasons as ultimately groundless may provide further support, from a different philosophical angle, for Jaspers’s remark, which I approvingly quoted earlier, that ‘the whole of my rationality rests upon the basis of non-reason’. Perhaps, having ascended and kicked away the ladder, we discover from Wittgenstein that it had always been floating in mid-air.

Prichard (2000, 2011, 2015) suggests that Wittgenstein’s late epistemology is relevant to philosophy of religion, and should be applied to it in the form of a position called ‘Wittgensteinian Quasi-Fideism’. I have discussed this suggestion and its implications elsewhere (Bennett-Hunter forthcoming), voicing the reservation that Pritchard’s specification of the theistic proposition ‘God exists’ as the distinctive ‘hinge proposition of religious belief’ is unduly restrictive. The added complexities of Prichard’s Wittgensteinian Quasi-Fideism notwithstanding,^6^ Wittgenstein (1969, §§94, 156, 233, 262) suggests, in several passages, that there is a necessary commonality to people’s hinge commitments, and that a set of such commitments is integral to a whole shared picture of the world, against which rational disagreements take place. It is problematic for Wittgensteinian Quasi-Fideism that ‘God exists’ is not a proposition that adherents to non-theistic religions could accept, less still secular atheists. Even within the Christian tradition, as we have seen, straightforward denials of the existence of God and insistence that talk of God’s existence is ineluctably non-literal have been part of Christian theology since the ancient world of Pseudo-Dionysius. Clearly the involvement of a commitment to theism is not a sufficient condition for an intellectual position to count as a religious one.

I advance the alternative proposal that, if there is at least one religious hinge proposition, it will have to be one on which both theists and atheists could agree. To return to my theme, I venture the thought that such propositions refer to experiences of ineffability, the possibility and occurrence of which are conceded even by the most vocal atheists. Yet, although they can be interpreted in secular terms, such experiences are also often interpreted in a religious light, both theistic and non-theistic (Bennett-Hunter forthcoming). This inclusive proposal would repay further development and evaluation by philosophers of religion, for it is the path that Jaspers saw as necessary for the hoped-for reunification of theology with philosophy, which has been in question since Tertullian. Moreover, it has already received initial support, from a quite different philosophical perspective, in the form of Silvia Jonas’s (2016, 184) recent suggestion that ‘Self-acquaintance’ could serve as a ‘minimal metaphysics’ of religious ineffability on which both the theist and the atheist could agree. One thing is certain, however: the future of theology as a philosophically credible epistemic practice will depend, at the very least, on a willingness to engage in conversation with non-theistic traditions.

It will be most important for philosophers of religion to pursue these lines of thought, which suggest that some important discussions between theists and atheists have been cut short prematurely. For we may find that such continuing conversations have the power to transform the character of the interreligious encounter (which is often also intercultural encounter) in ways that the contemporary world urgently needs. It will be clear from the foregoing article how I think that the philosophical appeal to ineffability, with its implied critique of ontotheology, can guard the human experience of what Jaspers called ‘Transcendence or God’ from dogmatically religious, and otherwise superstitious, misinterpretations. It can perhaps therefore achieve what even as prominent an atheist as the late Christopher Hitchens (2010) acknowledged to be *the* great cultural task of the future. With Karl Jaspers, I am hopeful about the valuable future contribution of philosophy to this great cultural task: its ability to remove the poison of exclusive, dogmatic religious claims, to enable religious experience to be melted down into the reading of a cipher, and thereby to clear the overgrown path between Athens and Jerusalem.

## Footnotes

1 Cooper’s capitalization, which I adopt here, is intended both to convey Dilthey’s sense of *das Leben* as the ‘permanent subject’ of meaning and to rule out purely biological senses (Cooper 2005, 126).

2 Some well-known twentieth-century examples include Paul Tillich (1968 vol. 1, 227, 262–3, 301), Simone Weil (for a sympathetic contemporary account, see Williams (2007)), and John Macquarrie (1984, 186).

3 See n. 1, above. This existential phenomenological conception of meaning is set out, *inter alia*, by the early Heidegger (1962, 116).

4 Similar themes are present in Wittgenstein’s later work, which I discuss in the final section.

5 For this quasi-religious, ‘sacramental’ line of thought in Merleau-Ponty, see Kearney (2010). For the notion of ‘real presences’, see Steiner (1989, 1996).

6 For example, Pritchard (2012, 267–268) distinguishes between hinge commitments and an ‘*Über*-hinge commitment’, which allows him to account for apparent hinge disagreements without conceding the existence of hinge disagreements. Since hinge commitments just codify the *Über*-hinge commitment, he interprets apparent hinge disagreements as second-order disagreements about which specific propositions should be included in the set of propositions that are taken to codify the general, shared *Über*-hinge commitment. When one inquires whether Wittgensteinian Quasi-Fideism is true (i.e., whether there are *religious* hinge propositions), this is the very point at issue, for one is asking whether or not some religious proposition(s) should be included in the set of specific propositions that are taken to codify the *Über*-hinge commitment.
